# Recurrent Takotsubo Cardiomyopathy Secondary to Cyclical Vomiting Syndrome

**DOI:** 10.7759/cureus.60764

**Published:** 2024-05-21

**Authors:** Abeera Akram, Ahmed Kazi, Sana Hyder, Stephanie Saucier

**Affiliations:** 1 Cardiology, University of Connecticut, Farmington, USA; 2 Internal Medicine, University of Connecticut, Farmington, USA; 3 Cardiology, Hartford Hospital, Hartford, USA

**Keywords:** takotsubo cardiomyopathy (tc), recurrent ventricular tachycardia, depression prevention, cyclical vomiting syndrome, stress-related cardiomyopathy

## Abstract

We present a case of a young woman having recurrent admissions secondary to cyclical vomiting syndrome complicated with stress-induced cardiomyopathy/takotsubo cardiomyopathy (TC). She not only had left ventricular dysfunction but also suffered from anxiety and post-traumatic stress disorder for which professional help was sought.

TC is defined as reversible, transient ventricular dysfunction in the absence of coronary artery disease. Due to the similarity of TC to acute coronary syndrome, TC is often left as a diagnosis of exclusion as it relies heavily on diagnosis by history, physical examination, and ultrasound imaging. Extreme emotional or physical stress can act as a trigger and timely identification and management of triggers causing TC are important to improve the outcome. In addition to physiological impact, TC also puts a toll on psychological health. Although the mechanism is not completely understood, reportedly plasma levels of epinephrine and norepinephrine were significantly elevated in patients with TC which might contribute to depression, anxiety, and psychological distress. Along with proper medical care, psychological care is equally important for patients with TC.

## Introduction

Takotsubo cardiomyopathy (TC) is a form of non-ischemic, reversible cardiomyopathy which predominantly affects women [1]. It is divided into typical and atypical. Typical TC is characterized by left ventricular dysfunction with regional wall motion abnormalities involving apex or apical ballooning, whereas atypical TC is characterized by akinetic/dyskinetic basal and mid-wall segments. TC is a diagnosis of exclusion, in the absence of angiographically significant coronary artery disease or acute plaque rupture. Etiology of TC is not fully understood but is always associated with an identifiable trigger. The trigger can be physiological (trauma, burns, or acute medical illness) or emotional (financial loss, death of a loved one, etc.) [2].

In our case, cyclical vomiting syndrome (CVS) acted as a trigger of recurrent TC. It is a rare trigger that is mentioned by a very few case reports [3]. CVS is defined as recurrent episodes of nausea and vomiting without any identifiable pathology [4]. It can last from hours to days. Patients undergo an extensive unrevealing workup over the course of months or years. This can lead to mental health disturbances. 

## Case presentation

A 38-year-old female with a significant past medical history of CVS and cardiac arrest secondary to long QT (KCNH2 gene variant) status post subcutaneous defibrillator (SubQ ICD) presented with chest pain. She was hemodynamically stable on presentation. On physical exam, the patient appeared in distress. She had a normal cardiac and pulmonary exam. Cardiac workup at the time of her initial cardiac arrest was unrevealing for additional cardiac abnormality. Prior to admission, she was being treated with beta blocker and metoclopramide chronically.

**Figure 1:**
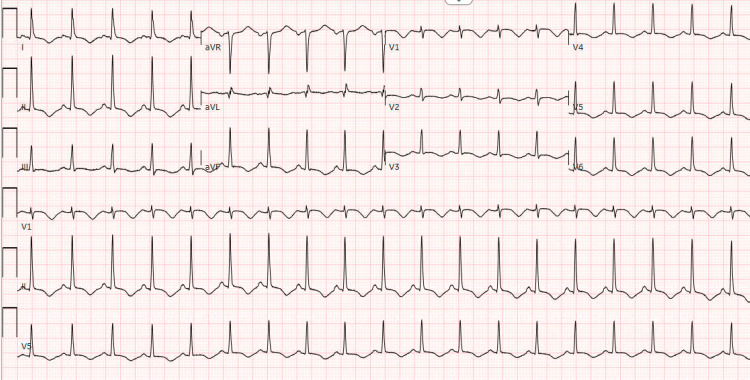
Electrocardiogram on presentation.

Upon presentation, she was given famotidine, dicycloverine, and promethazine for her vomiting. She was given sublingual nitroglycerin and hydromorphone for her severe chest pain with minimal improvement. Initial lab work revealed elevated hs-troponin of 113 ng/L (normal range <15 ng/L) and electrocardiogram (ECG) demonstrated T wave inversion in anterolateral leads (Figure 1). The patient was started on a heparin drip and transferred to a tertiary care center for further management and concern of non-ST elevation myocardial infarction.

**Figure 2:**
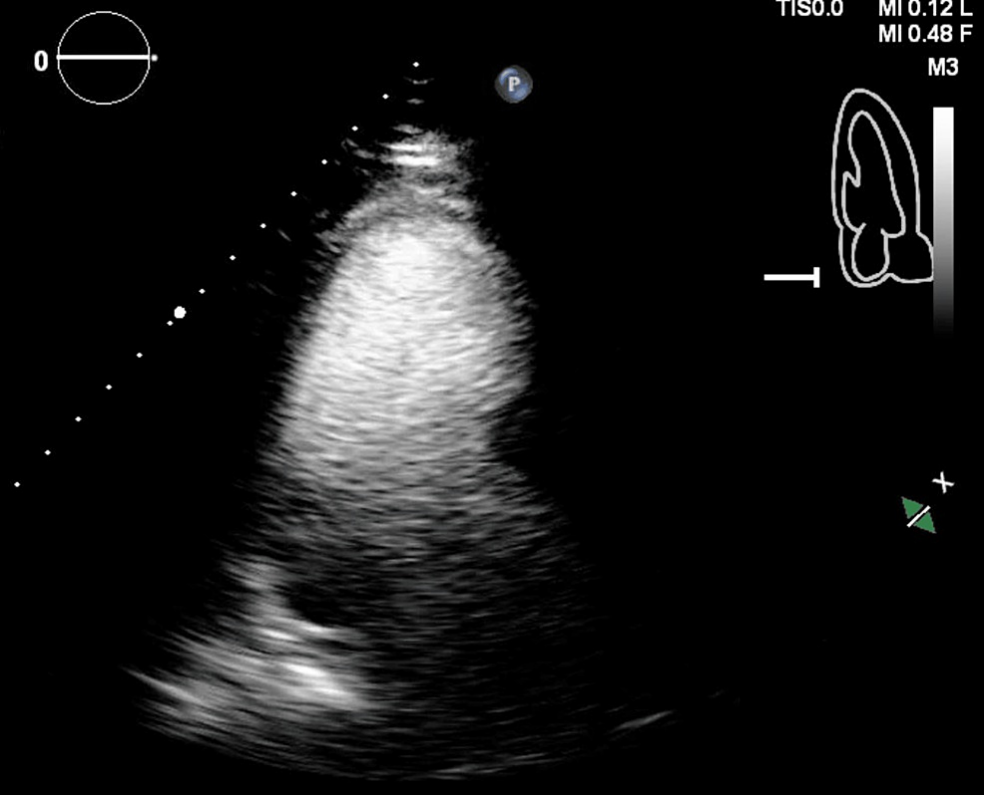
Initial echocardiogram showing apical ballooning.

An echocardiogram was performed revealing a left ventricular ejection fraction (LVEF) of 33% with regional wall motion abnormalities including apex, distal anteroseptal, and distal lateral wall akinesis with apical ballooning (Figure 2).

Based on her initial presentation and echocardiographic findings, the main differential diagnosis was TC. Since patients with TC commonly present with symptoms similar to those of obstructive coronary artery disease, initial diagnosis and treatment remain challenging. Acute coronary syndrome was also a differential so coronary angiogram was performed which revealed normal coronary arteries. In the setting of persistent chest pain, she was started on colchicine for the concern of pericarditis. Guideline-directed medical therapy (GDMT) was introduced for heart failure with reduced ejection fraction including angiotensin-converting enzyme inhibitor and mineralocorticoid antagonist, in addition to beta-blocker. A repeat echocardiogram was ordered to evaluate left ventricular function.

The repeat echocardiogram four weeks after presentation showed improvement in LVEF to 61% with resolution of wall motion abnormalities. Ten months later, she re-presented with a complaint of chest pain following cyclical nausea and vomiting episodes. An echocardiogram on admission revealed a severely decreased LVEF (20-24%) with regional wall motion abnormalities involving akinesis of the mid to apical segments and apical ballooning. She was started on a heparin drip and scheduled to have a repeat cardiac catheterization. Angiogram again showed no significant coronary artery disease. The hospital course was complicated by torsades de pointes in the setting of long QT, hypokalemia (3.0mmol/L), and hypomagnesemia (1.6mmol/L). She received three rounds of defibrillation from her ICD. The patient’s electrolytes were repleted.

She was started on lidocaine drip, treated with magnesium, and ultimately transitioned to mexiletine. The patient remained stable and was discharged on oral potassium and magnesium supplements in addition to GDMT and mexiletine. A repeat echocardiogram was performed eight weeks later with improvement in LVEF to 62% and resolution of wall motion abnormalities.

## Discussion

Recurrent TC is uncommon with a reported frequency of 1-6% of patients with TC [5]. TC results in transient left ventricular dysfunction and regional wall motion abnormality in response to an intense emotional or physical trigger. It is a diagnosis of exclusion requiring nonobstructive or absent coronary artery disease. Risk factors for recurrence include old age, male sex, diabetes, and chronic kidney disease. In-hospital mortality rates have ranged from 0 to 8% (4.1% in the International Takotsubo Registry study). Patients may develop signs and symptoms of heart failure, arrythmias, thromboembolism, or sudden cardiac arrest. In the InterTak registry, the rate of ventricular tachycardia (VT) was reported at 3.0% in patients with TC [6].

TC is often predisposed by a trigger [1], which in this case was CVS. CVS is a chronic functional gastrointestinal disorder characterized by nausea and vomiting lasting for one to five days followed by asymptomatic periods. In our patient, TC in addition to severe electrolyte disturbances in the setting of CVS, resulted in prolongation of her QTc and VT. Consequently, our patient started to experience post-traumatic stress disorder with symptoms of anxiety and nightmares.

The literature review shows association between TC and psychiatric disorders [7]. Not well proven but exaggerated sympathetic and catecholamine response is thought to be a central cause [8]. Depression tends to fluctuate between 20.5% and 48% and the prevalence of anxiety disorder is between 26 % and 56% of patients with TC. We have learned that besides good medical care, psychotherapeutic support is also warranted for patients with initial or recurrent TC. Our patient was discharged on oral potassium and magnesium supplementation, beta blocker, mexiletine, and anti-emetics including promethazine and cyproheptadine and had close follow-up with a cardiologist. In addition to her medical care, she has been following up with a psychiatrist to manage her anxiety and fear of having recurrent TC events.

## Conclusions

Recurrent TC is a rare phenomenon but usually, patients with the first episode of TC are at higher risk of recurrence. Recurrent episodes put a toll on patient's mental and physical health. Timely identification and appropriate management of triggers causing TC are important to improve the outcome. 

Our article emphasizes that in addition to proper medical care, psychological care is equally important for patients to prevent the recurrence of TC. Reporting case studies about various triggers helps in expanding our knowledge of rare conditions like TC.
